# Assessing Unmet Need for Mental Healthcare Among Adults in Germany – Results from a Nationwide Study with Data Linkage

**DOI:** 10.3389/ijph.2026.1608979

**Published:** 2026-06-11

**Authors:** Diana Peitz, Felicitas Vogelgesang, Heike Hölling, Thomas G. Grobe, Timm Frerk, Ursula Marschall, Julia Thom

**Affiliations:** 1 Department of Epidemiology and Health Monitoring, Robert Koch Institute, Berlin, Germany; 2 Department of Health Monitoring and Biometrics, aQua-Institute, Göttingen, Germany; 3 BARMER Institute for Health Care System Research (bifg), Berlin, Germany

**Keywords:** unmet need, mental healthcare, public mental health, mental health surveillance, patient perspective

## Abstract

**Objectives:**

To estimate the prevalence of self-reported (un)met need for mental healthcare and associated barriers among adults in Germany and to compare this information with documented mental healthcare use in order to assess the suitability of this indicator for mental health surveillance.

**Methods:**

Self-report survey and routine data documented by healthcare providers from 6,558 randomly sampled adults insured with a major German health insurance company built the basis of bivariate and multivariate analyses, also examining influences of sociodemographic determinants and mental health literacy. Prevalence estimates were additionally replicated with representative data from two national health surveys (n = 10,676 and n = 27,102).

**Results:**

57% of individuals with perceived need reported no mental healthcare use in the previous 12 months. Unmet need was associated with younger age, but not with sex or education. Most individuals with an unmet need reported internal barriers in terms of low mental health literacy. Self-report corresponds with documented mental healthcare use.

**Conclusion:**

Monitoring self-reported met and unmet need can inform healthcare planning from a patient perspective and addresses the mental health treatment gap.

## Introduction

Mental health problems contribute to the highest burden of disease worldwide, particularly when untreated [1, 2]. Therefore, monitoring population mental healthcare need is crucial for *mental health surveillance* (MHS). This information can be used by stakeholders to plan, initiate and evaluate actions improving populations’ access to care if needed [3].

In Germany, health insurance is mandatory to ensure care regardless of social status, gender or education. Mental healthcare is therefore accessible to everyone, at least in principle, as it is covered by statutory health insurance (SHI) and, depending on the rate, also by private health insurance companies (which insure 10% of the German population) [4]. Although mental healthcare is more accessible than in many other high-income countries, a considerable ‘*treatment gap’* can be assumed: Older representative data from 2009 to 2012 showed that the time between the onset of a mental disorder and the first contact to a mental health professional was six to seven years [5]. Moreover, only 24% of women and 12% of men diagnosed with any mental disorder had used either any general healthcare or specialized mental health services in the past 12 months [5]. Percentages vary depending on the diagnoses, for instance, only 16% of those persons with anxiety disorders were receiving specialized mental health services at that time. To be informed about the current state of mental healthcare need and its developments in the population, newer representative data is warranted. This data should ideally stem from multiple sources (patient-centered, SHI claims data, etc.) to avoid systematic measurement errors like over or underestimation through single-source bias and - in the long run - of longitudinal nature using the same measures to compare developments in the population over time. Nevertheless, the results emphasize, that mental health symptoms up to full blown disorders are no sufficient predictor for help seeking: Data from high-income countries shows that up to 51% of those with depression or anxiety do not perceive a need for professional help [6]. Even if a perceived need was reported, only 40% of individuals with an anxiety disorder reported lifetime use of specialized mental healthcare services due to their disorder in a representative German sample [7]. The other 60% can be considered person with “unmet need.” Unmet need can be used as an umbrella term for the phenomenon described above, i.e., “when someone has a mental health problem but does not seek or receive mental healthcare” [8]. Olsson and colleagues offer a more elaborated definition of this phenomenon and propose, that unmet needs can take place at three points of the way to sufficient care: 1) not perceiving a need for care though mental health symptoms are present, 2) not seeking care, and 3) not receiving (sufficient or adequate) care [9]. To learn more about specific barriers to mental healthcare along this path might be an important contribution to close the treatment gap and enable appropriate care.

In 2023, about one-quarter of the respondents across the European Union and in Germany in particular report that either they themselves, or a family member have encountered one or more barriers accessing mental health services [10]. Common barriers can be found on individual level in terms of low *mental health literacy* (MHL) meaning “knowledge and beliefs about mental disorders that aid their recognition, management and prevention” [11] (e.g., the assumption that the problem would resolve by itself, fear of stigmatization, belief that the care would not help, or not knowing where to turn for help [7, 9]) but also in terms of limited personal resources (time constraints, family or work obligations) or financial strain (paying the bus, missing work/pay) [12]. Barriers may also be due to external reasons, such as problems with the mental health professional who did not recognize the respective condition, or antipathy, which impedes the development of a therapeutic relationship. Also external, more structural reasons are widely reported: Access barriers subsuming long waiting times or not finding an accessible mental health professional at all, particularly in rural areas, are mentioned as common problems causing the mental health treatment gap [13–15]. Moreover, current forecasts for Germany indicate an increased demand for mental health professionals with up to 27% growth until 2035. This is markedly higher than for other health professionals [16] and points to an even increasing risk of undersupply in the coming years due to this structural reasons [17]. Therefore, actual mental healthcare needs should be monitored closely in the next decades to enable an evidence-based healthcare planning.

To employ *Unmet Need* as an indicator within the regular surveillance of mental health in Germany (building up trend and longitudinal data in the future) and consecutively to derive implications for a future needs-based provision of mental healthcare, a two-item operationalization was used to address the three main study aims:

1. We aim to explore the prevalence of self-reported unmet need for professional help regarding mental healthcare among adults in Germany and examine the distribution of unmet need among population subgroups, also considering potential differences in MHL.
2. We want to examine the barriers to access care in the German population and among certain population subgroups.
3. We aim to describe and compare the self-report of met and unmet need for professional help with regard to documented (mental) healthcare use according to statutory health insurance (SHI) claims data.

In line with the definition of Olsson and colleagues [9], we defined need for mental healthcare as *self-reported perceived need for mental healthcare*, and distinguished it from *unmet need* as refraining from seeking care although a perceived need is reported. Perceiving care as insufficient when seeking it was not assessed directly.

## Methods

### Participants

We used data from the German data linkage study *Optimized Data for Public Mental Health* (OptDatPHM) [18]. It contains survey data (paper and pencil questionnaire) from the last quarter of 2021 linked on person-level to SHI claims data from *n* = 6,558 participants randomly sampled among adults insured with one of Germany’s major statutory health insurance companies (BARMER). To respond to possible biases caused by selective participation, adjustment weighting according to the distributions of age, sex, region based on population statistics from 2020 [19] and education based on the Microcensus 2018 [20] was used across analyses. Four age groups were defined (18–29, 30–44, 45–64, 65+ years). A further separation of the oldest age group was not feasible due to small sample size of people with perceived need for mental healthcare in this age group, which build the basis sample for the main analyses. The CASMIN educational classification was applied to categorize participants’ level of education [21]. All sample characteristics can be found in Table 1.

**TABLE 1:** Sample characteristics (Data linkage study Optimized Data for Public Mental Health, Germany, 2021).

Characteristic	Overall sample		Perceived need		Unmet need of people with perceived need	
	% (weighted)	n	% (weighted) [CI]	n	% (weighted) [CI]	n
Total (N)	100%	6,558	**17.9%** [16.7–19.1]	1,053^a^	**57.3%** [53.7–60.9]	587
Sex (self-assessed)						
Female	51.1%	3,686	**21.2%** [19.7–22.9]	733	**58.3%** [54.0–62.4]	423
Male	48.9%	2,872	**14.4%** [12.7–16.2]	320	**55.9%** [49.2–62.3]	164
Age group						
18–29 years	16.0%	657	**31.6%** [27.6–35.9]	214	**65.4%** [57.4–72.7]	145
30–44 years	22.9%	1,032	**23.2%** [20.4–26.2]	261	**59.0%** [51.7–66.0]	162
45–64 years	34.8%	2,409	**17.0%** [15.4–18.7]	425	**51.0%** [45.6–56.3]	204
65 + years	17.8%	1,713	**6.0%** [5.0–7.2]	153	**49.2%** [40.3–58.2]	76
Level of education^b^						
Low	26.0%	1,509	**11.5%** [9.3–13.9]	130	**51.9%** [41.4–62.2]	70
Middle	57.0%	3,498	**19.9%** [18.3–21.6]	634	**59.2%** [54.6–63.7]	362
High	17.0%	1,497	**20.3%** [18.1–22.7]	272	**56.8%** [50.4–63.1]	151

^a^ 1053 of 6,491 valid cases, who answered this question.

^b^ In accordance with the CASMIN, classification system (see methods section).

CI = confidence interval.

In order to provide more robust evidence on the prevalence of unmet need for mental healthcare in the German population and population subgroups, we additionally calculated estimates with data from the representative telephone survey *German Health Update* 2021/2022 (GEDA) (*n* = 10,656 randomly sampled adults) [22]. Results for barriers to mental healthcare were replicated with data from the representative panel *Health in Germany 2024* (*n* = 27,102 randomly sampled adults, with online or paper-and-pencil questionnaire) [23].

### Measures

#### Self-Reported Unmet Need for Mental Healthcare

A two-item measure from the United States’ National Comorbidity Survey [24, 25] was used to assess *unmet need for mental healthcare* in the past 12 months. First, perceived need was assessed by the question 1: “*Was there ever a time during the past 12 months when you felt that you might need professional help because of problems with your emotions or nerves or your use of alcohol or drugs?*” (response options: “*yes*” or “*no*”) [26]. If affirmed, actual healthcare use was assessed by the question 2: “*Was there ever a time during the past 12 months when you have sought professional help because of problems with your emotions or nerves or your use of alcohol or drugs?*” (response options: “*Yes*” or ‘*No*’). *Unmet need* was defined if perceived need was reported (question 1: yes), but actual help-seeking was negated (question 2: no). *Met need* was assigned when both, perceived need and actual healthcare use were reported (question 1 & 2: yes). This sequence of the questions is orientated to the CIDI’s service use section [27] and therefore in line with former German representative data from 2009 on prevalence, impairment, and help-seeking for mental disorders [7].

#### Self-Reported Barriers to Mental Healthcare

Persons were additionally asked which barriers prevented them from getting professional help for their mental problems using a list of 12 possible barriers (response options: ‘*yes*’ or ‘*no*’, multiple answers possible) based on the CIDI’s service use section [27]. These questions on barriers were initially only intended for persons reporting unmet need, since the question began with: “If you wanted to seek help but have not done so: Which statements apply to you?”. As there were multiple answers from people with met need, we decided to report them as well and answers from persons with met and unmet need were compared in the end (see Table 3). Barriers were additionally subsumed following previous categorizations [7]: External reasons in terms of *Access Barriers* (i.e., unavailability; #1,2,3,4,8), external reasons in terms of *Provider-related Barriers* (i.e., problems with professional; #5,6,7) and, internal reasons in terms of *MHL-related Barriers* (i.e., self-reliance, perceived ineffectiveness of treatment, afraid of hospitalization, stigma; #9,10,11,12).

#### Mental Health Literacy

Due to its close relationship with help-seeking, MHL was entered as a covariate. The Mental Health Knowledge Schedule (MAKS [28]) was used to assess ‘*stigma-related mental health knowledge’* areas (6 items: help seeking, recognition, support, employment, treatment & recovery) and ‘*knowledge of mental illness condition’* (6 items: depression, stress, schizophrenia, bipolar disorder, drug addiction, grief) [28] with higher scores indicating higher knowledge and literacy. Additionally, a sum score comprising four items (“A mental illness is not a real medical illness,” “People with a mental illness could just snap out of it if they wanted,” “If I had a mental illness I would not tell anyone”, “If I had a mental illness, I would not seek help from a mental health professional”) from the Mental Health Literacy Scale (MHLS [29]) was used to depict further stigma-related attitudes toward mental disorders with higher scores indicating less stigma-related attitudes.

#### Documented (Mental) Healthcare Use

To describe and compare the self-report of met and unmet need for professional help with regard to documented (mental) healthcare use as documented in SHI claims data, four disjunct categories according the specificity and intensity of the mental health treatment were defined (see Table 4 for definition based on contact to selected physicians and psychotherapists, hospital diagnoses and billing codes): A) care by a *mental health professional* in an ambulatory setting or in a hospital setting, B) *psychosomatic basic care only* without care by a mental health professional according to A); C) *general healthcare only* with neither care according to A) or B); and D) *neither mental healthcare nor psychosomatic basic care nor general healthcare* according to A), B) and C).

### Statistical Analyses

We calculated percentages with 95% confidence intervals for people with perceived need for mental healthcare in general and separately for people with *met* vs. people with *unmet need*. This procedure was conducted for the total sample and within subgroups by sex, age, and level of education (study aim 1) and subgroups according to documented (mental) healthcare use (study aim 3). In addition to these descriptive data, a logistic regression model was calculated to prove for potential differences of sex, age and level of education (study aim 1, Table 2, model 1). In a second step, MHL was added to the model (Table 2, model 2) to prove whether there were additional group differences in MHL between people with met vs. unmet need. Barriers were listed as percentages with 95% confidence intervals individually and grouped in the three aforementioned reasons. Due to small sample sizes for the single barriers, only the three main categories of barriers were analyzed with logistic regression models to control for differences in sex, age groups, and education and in a second step for MHL (study aim 2). All analyses were computed with SAS 9.4 [30].

**TABLE 2:** Logistic regression: comparison of persons with met need vs. unmet need (Data linkage study Optimized Data for Public Mental Health, Germany, 2021).

Variable	Model 1		Model 2	
	Odds ratio	*p*-value	Odds ratio	*p*-value
30–44 years vs. 18–29 years	1.34 [0.84–2.12]	0.535	1.60 [0.98–2.61]	0.917
45–64 years vs. 18–29 years	1.85 [1.23–2.78]	0.035	1.94 [1.25–3.02]	0.081
65+ years vs. 18–29 years	1.82 [1.08–3.08]	0.176	1.96 [1.05–3.68]	0.277
Male vs. female	1.04 [0.75–1.44]	0.817	1.20 [0.85–1.71]	0.304
Level of education middle vs. low	0.80 [0.49–1.31]	0.335	0.59 [0.34–1.04]	0.282
Level of education high vs. low	0.88 [0.52–1.50]	0.937	0.52 [0.28–0.96]	0.067
​	​	​	0.96 [0.92–1.01]	0.106
MAKS - knowledge of mental health conditions
MAKS - stigma-related mental health knowledge	​	​	1.13 [1.01–1.21]	<0.001^a^
MHLS – stigma-related attitudes toward mental disorders (4 items)	​	​	1.23 [1.14–1.33]	<0.001^a^
Adjusted generalized R-Square	0.02	​	0.14	​

^a^ significant (<0.001).

## Results

### Self-Reported Unmet Need for Mental Healthcare

Perceived need for mental healthcare was reported by almost one-fifth of the total sample (18%, *n* = 1,053) (Table 1). Of these persons, more than half (57%, *n* = 587) reported no actual healthcare use indicating an unmet need for mental healthcare. For the total weighted sample, that means that 9% showed a need for professional help which was not met. The representative GEDA data showed comparable results (14% perceived need for mental healthcare; 59% of those reported unmet needs, accounting for 10% of the total weighted sample). The logistic regression models (Table 2) showed no significant effects for sex or educational attainment between people reporting met versus unmet need but there was a significant age effect (overall effect: *p* = 0.019) with higher percentage of met need for people aged 45–64 years than those aged 18–29 years (model 1). The likelihood of reporting unmet need declines with age (see Table 1). When entering MHL as a covariate (Table 2, model 2) significant correlations between higher MHL and the probability of reporting met need were observed: People with met need showed better stigma-related mental health knowledge and less stigma-related attitudes toward mental disorders.

### Self-Reported Barriers to Mental Healthcare

In general, internal reasons were reported more often than external reasons (see Table 3). The most frequent barriers named by individuals with unmet need were “wanted to deal with the problem on my own” (71.8%, CI: [67.6–75.9]) and “did not believe that a treatment would help” (27.4%, CI: [23.0–31.8]). External reasons were mostly access barriers (long waiting times/no provider found) and named by less than one-fifth (18.6% [14.9–22.3]/16.9% [13.4–22.3]) of those with unmet need. Representative data showed comparable results (see Appendix, Table A1).

**TABLE 3:** Barriers to mental healthcare (Data linkage study Optimized Data for Public Mental Health, Germany, 2021).

#	Barriers (ordered by reasons)	Total Perceived need (*n* = 1,053)		Unmet need (*n* = 587)		Met need (*n* = 464)	
		*n*	%	*n*	%	*n*	%
​	**Internal reasons: MHL-related barriers** ^a^	505	50.4	448	77.5	57	14.2
9	**I wanted to deal with the problem on my own**	**447**	**45.3**	**407**	**71.8**	**40**	**9.9**
10	**I didn’t think treatment would help**	**172**	**18.9**	**145**	**27.4**	**27**	**7.6**
12	I was worried about what people would think if they found out I was in treatment	110	13.2	96	19.3	14	5.1
11	I was afraid of being admitted to a hospital against my will	55	6.3	36	7.6	19	4.6
​	**External reasons: Access barriers (unavailability)**	285	27.6	215	35.1	70	17.7
3	**waiting time too long**	**150**	**15.9**	**109**	**18.6**	**41**	**12.3**
**1**	**did not find a practitioner/therapist**	**136**	**12.9**	**104**	**16.9**	**32**	**7.7**
2	did not get an appointment	114	11.6	88	15.2	26	6.8
8	Problems with reaching the location or scheduling	84	8.1	71	12.1	13	2.9
4	Problems with health insurance	20	1.7	13	1.6	7	1.8
​	**External reasons: Provider-related barriers (problems with professional)**	66	5.9	34	5.4	32	6.7
5	Practitioner saw no need/possibility to treat me	32	2.9	22	3.4	10	2.2
7	I did not like the practitioner	27	2.8	11	2.1	16	3.9
6	Practitioner did not take enough time	21	2.1	7	1.7	14	2.7

^a^ self-reliance, perceived ineffectiveness of treatment, stigma, afraid of hospitalization. MHL, Mental Health Literacy. The four main barriers are displayed in bold. Multiple responses were allowed.

Slightly more men (80.9% [74.4–87.4]) than woman (75.4% [70.6–80.1]) reported internal reasons and men (29.6% [21.8–37.5]) reported a few less access barriers than woman (38.4% [33.1–43.8]). However, results of the logistic regression model showed no significant differences by sex, age or educational attainment in reporting internal reasons (p_sex_ = 0.155, p_age_ = 0.754, p_educ._ = 0.617), access barriers (p_sex_ = 0.062, p_age_ = 0.722, p_educ._ = 0.718) or in provider-related barriers (p_sex_ = 0.714, p_age_ = 0.482, p_educ._ = 0.959). When adding MHL to the model, only the four MHLS items showed a significant correlation: Less “stigma-related attitudes towards mental disorders” (increased MHLS items) were correlated with reporting less internal reasons (*p* < 0.001) and more access barriers (*p* = 0.007).

In our study, also those with met need reported barriers in seeking professional help but less frequently and in the opposite direction: Persons with met need proportionally reported more external reasons in terms of access barriers than internal reasons in terms of MHL-related barriers than those with unmet need. For example, on the level of single barriers, only 10% of those with met need reported self-reliance in comparison to 72% of those with unmet needs.

### Self-Reported Met and Unmet Need Stratified by Documented (Mental) Healthcare Use

Table 4 shows the frequency of different types of care (ranging from no service use to specialist mental healthcare) in the four groups: overall sample, individuals with perceived need, individuals with met need, and individuals with unmet need. Self-reported met and unmet need revealed plausible discrepancies with regard to documented healthcare use: Most individuals with met need (70.6%) had contact with A) a *mental health professional*, whereas more than three-quarter (77.2%) with unmet need had no mental health-related care at all according to SHI data (added lines C & D).

**TABLE 4:** Self-reported perceived, met and unmet need by documented (mental) healthcare use (Data linkage study Optimized Data for Public Mental Health, Germany, 2021).

Visit of	Overall sample (*n* = 6,558)	Perceived need (*n* = 1,053)	Met need (*n* = 464)			Unmet need (*n* = 587)		
	%	%	%	[CI]	n	%	[CI]	n
A) *Mental Health Professional* ^a^	11.5%	34.7%	70.6%	[65.4–75.8]	330	7.9%	[5.3–10.5]	47
B) *Psychosomatic Basic Care only* ^b^	11.6%	13.4%	11.11%	[7.9–14.3]	56	15.0%	[11.8–18.2]	96
C) *Primary Healthcare only* ^c^	67.5%	34.7%	16.5%	[12.0–21.1]	70	64.3%	[59.6–68.9]	381
D) *Neither A) nor B) nor C)* ^d^	9.4%	8.1%	1.8%	[0.4–3.1]	8	12.9%	[9.3–16.4]	63

CI, confidence interval (to improve readability only shown for met and unmet need).

^a^ A) *mental health professional* in an ambulatory setting (defined as contact to a psychiatrist, child and adolescent psychiatrist, neurologist, medical or psychological psychotherapist, child and adolescent psychotherapist or specialist in psychosomatic medicine) or in a hospital setting (defined as main diagnoses of a mental disorder in inpatient or outpatient hospital treatment).

^b^ B) *psychosomatic basic care only* (defined as provision of services like differential diagnosis, verbal intervention for psychosomatic conditions and relaxation techniques provided by trained general practitioners (GP) or others than mental health specialists, documented by the accounting codes EBM 35100, 35110, 35111, 35112, 35113 and 35120) without care by a mental health professional according to A).

^c^ C) *primary healthcare only* (defined as contact to a GP) with neither care according to A) or B).

^d^ D) *neither care by a mental health professional nor psychosomatic basic care nor primary care* according to A), B) and C).

Additional exploratory analyses (not contained in Table 4) showed that half of those with met need received psychopharmacotherapy (52,4%). Psychotherapy was received by approximately one-third (30%) of them. In comparison, these treatments were much less commonly documented for person with unmet need: Less than 1% received psychotherapy (0.5%) and only 10% had documented psychopharmacotherapy. Approximately 5 out of 6 people with met need who only receive medication were in additional contact with a mental health professional (29.7% vs. 6.4%). On the contrary, 4 out of 5 people with unmet need who only take medication received it exclusively without a contact to a mental health professional (7.8% vs. 2.2%).

## Discussion

The present study aimed to 1) estimate the prevalence of unmet need for mental healthcare, 2) analyze the barriers to accessing mental healthcare, and, 3) describe and compare self-reported met and unmet need for professional help in relation to documented (mental) healthcare use.

### Self-Reported Unmet Need for Mental Healthcare in the German Adult Population

Almost one-fifth of the study sample reported a perceived need for mental healthcare. Of these, nearly 60% had an unmet need, which corresponds to one-tenth of the total weighted sample. These estimates could be confirmed by a representative German sample. Our findings are in line with older representative German data [7, 31] and international findings [6, 32, 33], which shows that the majority of individuals have unfulfilled mental healthcare needs.

Interestingly, no significant differences in unmet mental healthcare need were observed based on sex or educational attainment. This may be attributed to our methodological distinction between perceived need and unmet need, which is based on a staged help-seeking framework presented by Ollson and colleagues [9]. In contrast to unmet need, we observed sex-and education-related differences in perceived need in our previous work: women reported slightly higher perceived need than men when mental health symptoms were present, and perceived need decreased with lower educational attainment, despite the symptom burden being higher in this group [26]. Comparing the results between perceived need and unmet need might suggest, that strengthening need-oriented mental healthcare utalization in men and individuals with lower educational attainment may not primarily require tailored programs to promote care uptake. Instead, it may necessitate programs that assist these groups in accurately identifying the need for professional help when mental health symptoms are present, for example, through the selective promotion of MHL. Furthermore, this comparison underscores the importance of distinguishing between perceived need and unmet need in MHS to correctly interpret the data and to appropriately initiate, plan, and evaluate related public health interventions.

However, the likelihood of unmet need appears to decline with age, while barriers accessing care did not differ across age groups. This corresponds with lower administrative prevalence (indicating contact to a health professional) in younger age groups [34]. At the same time, epidemiological findings show that mental health disorders are more prevalent in the younger populations [35–37]. These results are consistent with findings on perceived need in this and previous work [26] which suggests that younger individuals also perceive a higher need for mental healthcare. Recently, however, the administrative prevalence of depression [34] and anxiety disorders [38] has increased more sharply in young adults than in older age groups. Moreover, mental health seems also to play a more prominent role in public discourse and social media among young people. Consequently, a reduction in the age gap for unmet need may be expected in the future.

### Self-Reported Barriers to Mental Healthcare

Analyses of barriers revealed that most individuals with an unmet need for mental healthcare reported internal barriers to mental healthcare in terms of low MHL: Although they perceived a need for professional help, nearly three quarters wanted to solve their mental health problems on their own and one-quarter considered treatment to be ineffective. This attitude or expectation seems particularly problematic as perceived need for professional mental healthcare has been shown to be strongly associated with significant psychopathology and functional impairment [26]. Access barriers, such as long waiting times or unavailable providers, played a secondary role. These findings align with older German [7] and international [39] data. Similar results were also found in an independent cohort study in Germany, where attitudes such as problem denial and help-seeking stigma emerged as the most prominent barriers among young adults, while structural barriers, such as long waiting times, were less significant [40]. Interestingly, public debates primarily focus on access barriers; for example, Eurobarometer data identifies long waiting times as the main reason for unmet mental healthcare needs (67% in the EU, 81% in Germany) [10]. However, MHL-related factors, such as stigmatization, were not assessed in this survey. Our logistic regression results highlight MHL as a significant predictor of not seeking professional help, underscoring the importance of including it as a key variable in future research to better understand its influence.

Inidividuals who report using professional help (met need) also encountered barriers to seeking help, but less frequently. While those with unmet need primarly reported internal barriers related to MHL-related barriers, the effect was reversed in those with met need, who more often reported external barriers related to unavailability of services. This is plausible, as individuals seeking professional help are less likely to hold negative attitudes but may still face barriers during referral processes or when attempting to access specific therapy. However, the comparison between individuals with met and unmet need further highlights that MHL-related, internal factors play a crucial role from preventing individuals from seeking professional help altogether. Legitimate concerns about ineffective treatment, discrimination, or compulsory care should be addressed during the treatment process.

### Self-Reported Met and Unmet Need Stratified by Documented (Mental) Healthcare Use

Self-reported met and unmet mental healthcare needs differ in a plausible way with regard to documented (mental) healthcare utilization, as indicated by billed services and their intensity and specificity according to SHI data. The proportion of individuals with met need who had documented contact with (A) *mental health professionals* was 9 times higher than that of individuals with unmet need (71% vs. 8%). P*sychosomatic basic care only (B)* was rare in both groups (11% vs. 15%), indicating only a slightly higher referral rate to mental health professionals for those with met need once mental health problems were assessed in general care for those with met need. At the same time, individuals reporting unmet need were more than four times as likely to be in (C) *general healthcare only* (64% vs. 17%) compared to those reporting met need, and six times as likely to contact (D) *neither general care nor mental healthcare* (13% vs. 2%)*.* Accordingly, the statement that they had not sought professional help predominantly reflects a lack of contact with specialized mental health professionals. For the remaining 8% who stated to have not sought professional help despite a visit by a mental health professional (A) was documented, it can be assumed that the services provided were not perceived as “professional help.” This may be the case, for example, if, after an initial contact (i.e., “Psychotherapeutic Consultation,” a mandatory service for German health providers to enable a low-threshold first contact with a psychotherapist to clarify treatment indication), specialist treatment was not considered indicated or because after this initial contact a treatment could not be offered due to a lack of care capacity [41, 42].

Explorative analyses pointed out that receiving psychotherapy was almost exclusively understood as healthcare use for mental health problems (reported by less than 1% among those with unmet need). The documented prescription of psychotropic drugs was more often associated with the report of no further, specialized healthcare use for mental health problems: The majority of the individuals with unmet need were not receiving additional specialist mental healthcare (e.g., face to face consultations where the being asked about their symptoms by a health professional) despite being prescribed psychotropic drugs while those with met need almost all reported a contact with a mental health professional in addition to the prescription of medication.

The deeper exploration of discrepancies between self-report data and SHI data is beyond the scope of this manuscript. However, the results in Table 4 show that individuals with met needs have a high documented use of specialized care: A) 70.6% and B) 11.1%. However, these numbers do not add up to 100%. This may be due to the fact that members of the general public might not be able to distinguish between evidence- and guideline-based treatments (e.g., psychotherapy, pharmacotherapy), GP consultations, and complementary and alternative medicine (CAM), and may consider the latter as “professional help” as well. Though, particularly information of CAM cannot be obtained from SHI data.

### Limitations

The following limitations should be considered: 1) The study is based on randomly sampled members of an insurance company comprising about 10% of the German population. However, weighting minimized demographic differences to the general population. Replicating key analyses in a general population sample strengthens the findings’ validity. 2) The assessment of unmet needs in this study was based on the approach used in the United States’ National Comorbidity Survey [24, 25] and orientated to the CIDI’s service use section [27], thus enabling an international comparison of the need for assistance. However, due to the economical question design for surveillance purposes, it is only a brief inquiry into (un)met needs. Consequently, the categories for the reasons help is required have been consolidated. In a more elaborated survey on unmet needs, categories for the reasons for help seeking or the response categories could be more extensively defined (e.g., “don’t know”), in alignment with more elaborated research question. 3) Moreover, the barriers assessed in this work are based on the CIDI’s service use section [25] as well and are additionally subsumed under previous categorizations [7] from German population studies to facilitate national and international comparisons. However, important individual/personal reasons are missing, such as limited personal resources (time constraints, family or work obligations) or financial strain (e.g., paying for transportation, missing work/pay) [12]. These barriers should be included in future surveillance work on need barriers at the population level. 4) Self-report measures may be affected by social desirability and recall biases.

### Conclusions

Over half of individuals with a perceived need for mental healthcare remain untreated, accounting for approximately 10% of the population. Barriers are predominantly internal and linked to low mental health literacy (MHL), rather than structural access barriers. Closing the mental health treatment-gap should involve the appropriate promotion of MHL, including fostering an accurate understanding of when professional help is indicated, enhancing recognition of primary care as the initial access point within a stepped care model, and improving access to specialized mental healthcare services. The effectiveness of such approaches at population level can be assessed through continuous surveillance of unmet mental healthcare need using the validated self-report indicator presented here.

## Data Availability

Since informed consent from OptDat study participants did not cover the public deposition of data, the OptDat dataset generated and analyzed is not publicly available. Data from the German Health Update 2021/22 can be provided by the Robert Koch institute on reasonable request. Data from the panel Health in Germany 2024 will also be made available by the Robert Koch Institute during the course of 2026.

## Appendix

**TABLE A1:** Replication in representative survey data: barriers to mental healthcare (RKI Panel ‘Health in Germany’, Germany, 2024).

#	Barriers (ordered by reasons)	Unmet need (*n* = 2,977)	
		*n*	%
​	**Internal reasons: MHL-related barriers***	2,276	75.3
9	**I wanted to deal with the problem on my own**	**1977**	**65**
10	**I didn’t think treatment would help**	**621**	**21.9**
12	I was worried about what people would think if they found out I was in treatment	410	15.2
11	I was afraid of being admitted to a hospital against my will	131	6
​	**External reasons: Access barriers (Unavailability)**	1,148	37.8
3	**waiting time too long**	**505**	**18.3**
**1**	**did not find a practitioner/therapist**	**652**	**21.6**
2	did not get an appointment	417	14.2
8	Problems with reaching the location or scheduling	275	8.5
4	Problems with health insurance	67	2.0
​	**External reasons: Provider-related barriers (problems with professional)**	159	6.4
5	Practitioner saw no need/possibility to treat me	73	2.6
7	I did not like the practitioner	79	3.0
6	Practitioner did not take enough time	36	2.0

* self-reliance, perceived ineffectiveness of treatment, stigma, afraid of hospitalization. MHL, Mental Health Literacy. The four main barriers are displayed in bold.
